# Motivational Resources of Agency in Adolescents’ Career Development in Postsecondary Transition: More than Being Self-Efficacious

**DOI:** 10.1177/08948453251351576

**Published:** 2025-06-14

**Authors:** Jiseul Sophia Ahn, Catherine F. Ratelle, Stéphane Duchesne, André Plamondon

**Affiliations:** 1 School of Psychoeducation, Université de Montréal, Montreal, QC, Canada; 2 Faculty of Educational Sciences, Université Laval, Quebec, QC, Canada

**Keywords:** career decision-making self-efficacy, career decision-making autonomy, vocational identity, career exploration, postsecondary transition, person-oriented analysis

## Abstract

A sense of agency is crucial for self-directed career development, particularly during the postsecondary transition, a period shaping career trajectories. This study examined autonomy and self-efficacy as pillars of agency, using longitudinal data from a sample of 637 French-Canadian adolescents (54% girls, *M*_age_ = 16) before and after the transition. Participants reported on career decision-making autonomy (intrinsic, identified, introjected, and external motivations) and self-efficacy, along with career exploration and commitment. Latent profile analysis identified four subgroups of youth, including youth with high autonomous motivations and self-efficacy, displaying a fully agentic profile, and others with lop-sided agency, marked by strong controlled motivations. Relative weigh analysis indicated self-efficacy as the strongest predictor, with intrinsic motivation making a unique albeit weaker contribution to exploration. These findings highlight the importance of fostering both autonomy and competence, while also revealing the detrimental implication of reward- and punishment-driven motivation on career identity integration.

## Introduction

Life transitions bring multifaceted changes that prompt young people to reconsider who they are and the direction they want to take, making these periods fertile grounds for studying their career and identity development (Branje, 2022). Postsecondary transitions, in particular, entail moving beyond the structured environment of secondary school into a less scripted world, thus marking a decisive stage for employment prospects and long-term well-being in adulthood (Marttinen et al., 2018). This stage opens doors to a wide range of career pathways, such as entering the workforce or pursuing higher education in different fields. Although the increased flexibility and freedom can be empowering, without the right support and guidance, it may also feel daunting especially for young people who are still forming their sense of self and learning about the world of work (Christiaens et al., 2021). It is crucial to support youth during this period to help steer their career development toward fulfilling professional and personal lives. To inform such support strategies, a key priority for educational and counseling researchers and practitioners is to deepen our understanding of the factors contributing to career development: That is, what motivates youth to actively engage in career exploration and eventually commit to a personally meaning career path?

Exploration and commitment are goal-directed behaviors integral to career development and can be studied through a motivational lens. Many theories in vocational psychology, career development, and motivation converge on the assumption about the self as an agent: People are self-organizing, self-regulating, and proactive, actively shaping their life experiences, rather than simply reacting to them (Bandura, 2005; Hartung & Subich, 2011; Ryan & Deci, 2017). However, these theories diverge in their perspectives on the source of agency, specifically the motivational resources that underpin it. This study focuses on two prominent motivational theories: social cognitive theory, which emphasizes self-efficacy as the crux of human agency (Bandura, 2005), and self-determination theory, which, while recognizing the significance of perceived competence, places greater emphasis on perceived *autonomy* (Ryan & Deci, 2017).

Despite their theoretical importance, few studies have tested an integrative model of career development, often treating self-efficacy and autonomy as competing predictors. For example, variable-centered approaches like regression analyses have been used to determine which resource—between self-efficacy and autonomy—shows stronger predictive utility (Cordeiro et al., 2015; Guay et al., 2003, 2006). Although informative, such an approach falls short in examining self-efficacy and autonomy combine within an individual and what implications their interplay has for career development. Consider, for instance, two adolescents: one who feels both highly confident and autonomous, and another who feels confident but also controlled and pressured. Do these differences in motivational profiles lead to different career development outcomes? Is self-efficacy alone sufficient, or does perceived autonomy confer additional advantage in fostering agency?

By considering both self-efficacy and autonomy, this study aims to offer a more holistic and nuanced understanding of youth’s agency in career development. Specifically, using person- and variable-centered approaches, the study aims to (1) examine the independent contributions of these motivational resources to adolescents’ career development (e.g., variance explained by self-efficacy controlling for intrinsic motivation and vice versa), as was done in previous studies, (2) explore intraindividual configurations of these distinct motivational resources, and (3) examine their joint contributions to career development.

### Career Development Across the Postsecondary Transition: Exploration and Identity Commitment

Career development is a lifelong process of developing one’s vocational identity through the iterative process of exploring and committing (Porfeli & Lee, 2012). Vocational identity refers to the sense of self at work (“who I am as a worker”). A well-developed identity provides a sense of direction and certainty about where one is going in life, particularly in coping with the challenges associated with life transitions (Branje et al., 2021; Erikson, 1968; Marttinen et al., 2018). Having a strong identity commitment is an important asset facilitating the postsecondary transition and effective functioning in university (Germeijs & Verschueren, 2007), whereas lack of commitment can endanger a steep increase in anxiety (Christiaens et al., 2021).

Career exploration is an indispensable process in career development (see for a review, Jiang et al., 2019), and in-depth (vs. in-breadth) exploration rises in significance as the developmental deadline of choosing a career is imminent as in the postsecondary transition (Porfeli & Lee, 2012). In contrast to in-breadth exploration (which involves “looking around,” considering diverse options), in-depth exploration involves “focusing on,” narrowing down to a smaller range of career options by assessing their fit with personal attributes of values, goals, skills, and interests (Gati & Kulcsár, 2021; Porfeli & Lee, 2012). The information and insights gained from this targeted exploration enable youth to consolidate their identity commitment (Bogaerts et al., 2019) and follow through with their career plans with greater confidence (Cheung & Jin, 2016; Germeijs & Verschueren, 2007). In-depth career exploration (referred to simply as exploration thereafter) and commitment are core components in the process-based view of career development, and served as the primary outcome variables in our study, which were examined using motivational perspectives of social cognitive theory and self-determination theory.

### Social Cognitive Career Theory: Career Decision-Making Self-Efficacy

Built on the core principles of Bandura’s social cognitive theory (Bandura, 2005) and adapted to the realm of career development, social cognitive career theory outlines how individuals’ cognitive self-perceptions, informed by social cues, predict an array of career-related behaviors, choices, and interests (Lent et al., 2016). Central to this theory is career decision-making self-efficacy (CDSE), defined as the confidence one has in their ability to carry out career development activities. Amongst many other social cognitive motivational constructs, CDSE is theorized to be the most proximal predictor of career behaviors and the primary force fueling youth agency in career development (Lent et al., 2016). That is, adolescents act like an agent when they demonstrate strong confidence in their ability to handle the complex tasks involved in career development.

CDSE is one of the most extensively studied motivational constructs in career development and vocational psychology. Meta-analyses have highlighted CDSE’s role in reducing career indecision (Choi et al., 2011; Udayar et al., 2020); longitudinal research has demonstrated that CDSE predicts longitudinal increase in exploration and commitment among high school students (Cordeiro et al., 2015; Lee et al., 2016), even when controlling for personality traits and demographic factors (Rogers & Creed, 2011). Moreover, intervention programs designed to increase CDSE in high school students have also been shown to promote career exploration (Chiesa et al., 2016), underscoring CDSE’s theoretical and practical relevance in supporting youth career exploration and commitment. However, self-efficacy alone may not always translate into motivation for an activity. It is possible for individuals to believe that they can successfully complete a task yet still lack the drive to engage in it. This gap in motivation is where self-determination theory offers valuable insights.

### Self-Determination Theory: Career Decision-Making Autonomy

In sync with social cognitive theory, self-determination theory (SDT; Ryan & Deci, 2017) acknowledges the importance of perceived competence, viewing it as one of the basic psychological needs for human development^1^. However, SDT places a stronger emphasis on the experiences of autonomy at the heart of human motivation. Autonomy is defined as the feeling of volition, willingness, and self-endorsement of one’s actions (Ryan & Deci, 2017). According to SDT, autonomy, along with competence, is one of the basic psychological needs that, when satisfied, energizes people’s innate growth-oriented tendencies toward psychological growth and integration. Beyond viewing autonomy as a need, SDT also conceptualizes it as a mode of functioning. That is, different reasons why people engage in a behavior can be mapped along a continuum of perceived autonomy, representing different quality—not just the quantity—of motivations.

Young people can approach career development with four different types of motivations that vary in relative autonomy (Guay, 2005). *Intrinsic motivation* involves engaging in an activity because of the inherent pleasure one gets from it. *Identified regulation* involves engaging in an activity because one sees the personal value in it. *Introjected regulation* involves engaging in an activity to protect the ego and to reduce internal pressures (e.g., guilt and anxiety). Lastly, *external regulation* involves engaging in an activity merely out of external contingencies (e.g., rewards and punishments). Throughout the paper, we collectively refer to intrinsic and identified motivations as autonomous forms of motivations, whereas the last two (introjected and external motivations), controlled, for conciseness. This grouping is based on both theoretical assertions and empirical evidence indicating the relative autonomy associated with each motivation type, their intercorrelations as well as links with outcome variables (Howard et al., 2017, 2021). Whereas integrated regulation (i.e., engaging in a behavior because it aligns with and expresses who they are) is a form of autonomous motivations, this form of motivation is empirically indistinguishable from identified regulation (Howard et al., 2017). Due to its assessment challenges, it was not assessed in the present study.

Several studies examined how the quality of motivations toward career decision-making related to young people’s career development. One study involving high school students found that autonomous (i.e., intrinsic and identified) motivations in career decision-making were linked to engaging in systematic and intentional career explorations, which helped reduce indecision, whereas controlled (i.e., introjected and external) motivations were linked to ruminative exploration that increased indecision (Paixão & Gamboa, 2021). Notably, those driven by controlled reasons were found to be less committed to their career choice, even after having engaged in similar levels of exploration as their autonomously motivated peers (Paixão & Gamboa, 2017). Similarly, a three-year study with college students revealed that those experiencing chronic indecision had lower levels of autonomy at the start of college compared to their more decided peers (Guay et al., 2006). Altogether, these findings suggest that youth engage in career development with either autonomous or controlled motivations, and that the sense of autonomy (or the lack thereof) has distinct and important implications for their career development.

### Bridging Two Theories: Self and Agency in Youth Career Development

The two schools of thought—the social cognitive theory and SDT—have both contributed significantly to our understanding of how to empower the self and create the conditions for optimal functioning and well-being. However, these bodies of research have largely developed in isolation, particularly within the fields of career development and vocational psychology. Despite some differences—such as the emphasis on self-efficacy versus autonomy as the central motivational factor, or the focus on the quantity versus quality of motivations—both theories share a core assumption about human nature: the agentic tendencies of the self.

Agency refers to intentionally acting upon one’s environment or personal functioning to bring about desired changes (Bandura, 2005; Chen & Hong, 2020). Transposed to youth career development, when young people feel agentic in their career choices, they experience a sense of ownership and authorship, feeling both capable (i.e., self-efficacious) and autonomous (i.e., motivated by personal values and interests) in directing their career path in line with their self-endorsed goals, values, and interests (Lent & Fouad, 2010; Soenens & Vansteenkiste, 2011). Although most career development theories highlight the importance of self-development and agency as a key criterion of optimal career development (Hartung & Subich, 2011), few studies explicitly examined the extent to which young people are *self*-directed in their career development—guided by their personal interests and values (except Waterman & Schwartz, 2024). Existing research on career development agency often focuses on perceptions of competence (e.g., Lent & Fouad, 2010), self-regulatory capacity (e.g., Chen & Hong, 2020) and aspirations (e.g., Descary et al., 2023), while paying scant attention to youth’s sense of autonomy as defined by volition and self-endorsement of their career goals, choices, and behaviors.

A handful of studies have attempted to integrate both self-efficacy *and* different types of motivations varying in degrees of autonomy as resources of agency in career decision-making. These studies have highlighted the proximal role of career decision-making self-efficacy in predicting greater career exploration and commitment (Cordeiro et al., 2015; Guay et al., 2003, 2006), with some studies reporting that autonomy contributed to explaining unique variance in career indecision, albeit more weakly than self-efficacy (e.g., Guay et al., 2003). However, these studies have used a single indicator of autonomy, aggregating the levels of four different types of motivations (Guay et al., 2003, 2006), or the general need satisfaction and frustration, which combines autonomy, competence, and relatedness needs (Cordeiro et al., 2015). This measurement strategy potentially obscures the unique consequences associated with each motivation type along the autonomy continuum, as highlighted in the studies mentioned above.

Moreover, these studies have primarily focused on university students after the transition (e.g., Guay et al., 2003, 2006) or high school students before the transition (Cordeiro et al., 2015). Further scrutiny is warranted to examine the long-term implications of these two key motivational precursors of career development, using a longitudinal study that repeatedly assesses youth’s career development before and after the transition. Lastly, none of the studies examined how the distinct motivational resources of self-efficacy and autonomy components combine within an individual. Autonomy and self-efficacy may not always co-occur: The correlations between CDSE and four types of motivations are low to moderate in size, with CDSE positively linked with autonomous motivations, but negatively with controlled motivations (Guay, 2005). This suggests that teens feeling competent about navigating the career decision-making activities may experience different levels of autonomy; some may feel very volitional because they are motivated by personal interests and values, whereas others feel controlled because they are complying with what their parents say or because they are motivated by anxiety or pride.

### The Present Study

What does it mean to be an agent in one’s career development? Social cognitive views identify self-efficacy or CDSE (the belief that “I *can* make my intention happen”) at the crux of agentic functioning, whereas SDT argues it is the feelings of autonomy (the perception that “I am doing it because it interests me and it’s important to me”). Aiming to bridge these two theories and to expand our understanding of motivational resources of agency, this study examined perceptions of both self-efficacy and autonomy as motivational precursors that catalyze youth’s in-depth career exploration and identity commitment during the postsecondary transition, a decisive period for youth’s career development. We used person- and variable-oriented approaches to examine the unique and joint contributions of feeling both efficacious and autonomous.

This study was conducted in the province of Quebec, Canada, whose educational system consists of five years of secondary school, followed by college (also known as CEGEP). French acronym for College of General and Professional Teaching, CEGEPs are public postsecondary educational institutions, enrolling as much as 70% of secondary diploma holders in Quebec (Ministère de l'Éducation, 2024). Students in CEGEP can opt for either a 3-year technical, professional program (offering a direct link to a job market through specialization in a specific career path, such as dental hygienist, social service technician, and engineering technician) or a 2-year pre-university program (offering a link to higher education like university for a bachelor’s degree). Given the decisiveness of this period for youth’s career development, this study focused on the two years surrounding this transition, the last year of secondary school and the first year of CEGEP.

We put forward three hypotheses. **First**, we expected different subgroups of youth characterized by distinct configurations of self-efficacy, autonomous motivations (intrinsic and identified), and controlled motivations (introjected and external) toward career decision-making. For instance, some would be “fully agentic” reporting high levels of self-efficacy and autonomous motivations, with lower levels of controlled motivations, while others would show “lop-sided agency” highly confident but less autonomous and more controlled. **Second**, we expected fully agentic youth would show greater exploration and a clearer identity, compared to those who show lower agency or a lop-sided profile of agency. **Third**, we expected the two autonomous motivations and self-efficacy to explain unique variance in exploration and identity commitment pre- and post-transition, with self-efficacy explaining greater variance. The two controlled motivations were expected to add no unique contribution or worse relate to lower exploration and commitment, above and beyond what is explained by self-efficacy and autonomous motivations. In testing these hypotheses, we accounted for sociodemographic variables (e.g., gender and socioeconomic status), and school grades, that have been shown to shape youth career development (Bryant et al., 2006).

## Method

### Data Source and Participants

We used an existing dataset from a research project that annually tracked 659 French-Canadian adolescents and their families for six years starting from 2011 when the adolescents were in Secondary 3 (equivalent to Grade 9). Participants in this project came from a government-issued stratified list of students enrolled in their third year of secondary school in the Canadian province of Quebec. Those who agreed to participate in the study electronically filled out online consent forms and questionnaires that were hosted on a secure university server. For this study, we used the data from 637 French-Canadian adolescents in Secondary 3 (54% girls; *M*_age_ = 14) who participated at least once in the first four waves (from Secondary 3 to 5, and Cegep 1). Whereas only data from Secondary 5 (Time 1 [T1]) and Cegep 1 (T2) were used in main analyses, we had a broader inclusion criterion to maximize the sample size and to use sociodemographic variables and measures of career development and school adjustment reported in earlier waves for missing data estimation (see Data analyses, Missing Data below). At the onset of the project (in Secondary 3), most of the participants were attending public schools (79%), living with two biological parents (65%), in the general academic track (90%), and from middle-class families: More than 90% of the parents had a high school diploma at least and the average family income was comparable to the provincial level at the time of the data collection (Statistics Canada, 2013). Majority were French speakers (94%) and born in the province of Quebec (93%). Of those with available information at T2 (*n* = 397), 79% reported being in college, 11% in secondary school, and 6% on the job market.

### Measures

#### Career Decision-Making Autonomy

At T1, adolescents completed the 32-item Career Decision-Making Autonomy Scale (Guay, 2005). Developed and validated in French, this multidimensional scale assessed four types of motivations by asking adolescents, to indicate on a 7-point scale, ranging from 1 (*does not correspond to all*) to 7 (*corresponds completely*), “why” they engage in 8 career decision-making activities (e.g., seeking information on careers and identifying a career option). The scale assessed (1) intrinsic motivation (e.g., “… for the pleasure of doing it”); (2) identified regulation (e.g., “… because I believe it is important”); (3) introjected regulation (e.g., “… because I would feel guilty and anxious if I did not do it”); and (4) external regulation (e.g., “… because someone is forcing me to”). Each motivation type showed high internal consistency (ωs = .91–.95) (see Section S1.1 in the Online Supplemental Materials for details on measurement models).

#### Career Decision-Making Self-Efficacy

At T1, participants completed 8 selected items from the Short Form of the Career Decision-Making Self-Efficacy Scale (Betz et al., 1996), which has been translated into French and previously used with the French-Canadian sample (Guay et al., 2006). They indicated how confident they felt they could complete the tasks described in each item on a 5-point scale ranging from 1 (*no confidence at all*) to 5 (*complete confidence*). Each item described a career decision-making task (e.g., making a plan of your goals for the next 5 years). The scale showed satisfactory internal consistency (ω = .85; see Section S1.2).

#### In-Depth Exploration

At T1 and T2, participants completed the Career Exploration Survey (Stumpf et al., 1983), translated and previously used in the sample population (Denault et al., 2019). They indicated how engaged they were in career exploration within the last 3 months using a 5-point scale ranging from 1 (*not much or not at all*) to 5 (*a lot*). Out of the 15 items in the original scale, 7 were selected based on the conceptual definition of in-depth exploration and were reviewed by two career development experts, both of whom were external to this study’s authorship. These items included “I initiated conversations with knowledgeable individuals in my career area” and “I reflected on how my past integrates with my future career” (see S1.4 for the full list of items and measurement models).

#### Vocational Identity Commitment

At T1 and T2, participants completed the 9-item in French translated from the Vocational Identity Scale (Holland et al., 1980), translated into French. They indicated how well each item described themselves on a 5-point scale ranging from 1(*does not describe me at all*) to 5 (*perfectly describes me*). Items included “I’m mixed up about this whole career choice issue.” The scores were reverse coded such that the higher scores indicated clearer and stronger vocational identity (see S1.5).

#### Sociodemographic Information

At the onset of the study, adolescents reported on their gender, age, and school type; while their parents provided information on their marital status, family income, and educational attainment. To parsimoniously account for the socio-familial factor, we calculated a socio-familial adversity index (SFAI), using the measures of family type, household income, and parental educational level (see S 1.6 for details). The majority of our sample (95%) was considered not at risk (SFAI = 0) or low at risk (SFAI = .33), whereas less than 1% (*n* = 4) participations was considered high at risk (SFAI = 1) and only 4% were considered moderately at risk (SFAI = .67).

#### School Grades

Adolescents self-reported their school grades in French and math in Secondary 3 to 5. These scores (with an average correlation of .57) were averaged to form a single composite score representing overall school performance, which ranged from 1 to 100.

### Data Analyses

All analyses were conducted in Mplus (Muthén & Muthén, 1998-2017) and in R using the MplusAutomation Package (Hallquist & Wiley, 2018).

#### Missing Data

Of 637 adolescents, 457 (71%) participated at either T1 or T2 (with 180 participating at both timepoints), whereas 180 (28%) participated at neither. For the latter group, only available data were sociodemographic information and school adjustment from earlier waves not included in this present study. To test if there are any differences between adolescents who participated either at T1 or T2 versus those did not at all, we conducted a multiple regression, where the group membership predicted an array of sociodemographic variables. The results indicated that adolescents who participated at T1 or T2 (vs. who did not) reported higher school grades (β = .13) and came more advantaged socio-familial background (β = .18), whereas showing no differences on age, gender, and school track. These variables (namely, age, gender, school grades, and SFAI) were controlled for subsequent analyses. To deal with missing data, we used the full information maximum likelihood (FIML) estimation with robust standard errors (the MLR option in Mplus 8.1), a gold-standard technique for missing data treatment that uses all available data to improve statistical power, while correcting for nonnormality (Enders, 2010; Graham, 2009). Also, to make the assumption of missing at random (MAR) more plausible, we incorporated auxiliary variables for missing data estimation using the principal component approach (Howard et al., 2015; see S2.1 for details).

#### Preliminary Analysis: Measurement Models

We tested measurement models for all study variables, as well as longitudinal invariance for variables measured at two waves, namely, exploration and commitment. We conducted confirmatory factor analyses and saved factor scores of the latent variables (mean of 0 and variance approximating 1), which were then used as indicators in subsequent main analyses (see Section S1 in the Supplemental Materials for more details). Unlike mean or sum scores, factor scores take into account the differential contribution that each item makes to capturing the latent factor (McNeish & Wolf, 2020). Factor scores of 0 represent the group-level mean, while greater than 0 means above-average endorsement of the construct (e.g., more confident and more intrinsically motivated).

#### Person-Oriented Approach: Latent Profile Analysis

Using factor scores of self-efficacy and four types of motivations varying in relative autonomy, a series of latent profile analyses (LPA) were conducted to identify the best profile solution. To avoid convergence on local maxima, all models were estimated using 5000 random starts, 200 iterations, and the 300 best solutions were retained for final stage optimization (Morin & Litalien, 2019). Means were freely estimated in all profiles, while constraining variances to be equal across profiles. The decision of the optimal number of profiles was guided by theoretical interpretations of the profiles and the model fit statistics (Bauer & Curran, 2004). Fit indices used to assess the quality of the profile solutions include the Akaike information criterion (AIC), the Bayesian information criterion (BIC), the sample size–adjusted BIC (ABIC), the adjusted Lo, Mendell, and Rubin likelihood ratio test (aLMR), and the Bootstrap likelihood ratio test (BLRT). A lower value on the AIC, AICC, BIC, and ABIC suggests a better fitting model, and a statistically significant *p* value on the aLMR and BLRT supports a model with one less profile. If these indicators keep decreasing without reaching a minimum, we inspected an elbow plot for a plateau (i.e., the point after which the slope flattens suggests the optimal number of profiles).

Once the final solution was chosen, the manual 3-step BCH procedure was used to compare the mean levels of exploration and identity across profiles, while controlling for SFAI, gender, and school grades. The BCH method is recommended when estimating profile-specific means for continuous distal outcomes, because (1) this method uses BCH weights to account for probabilities of profile classification and; (2) it prevents profile shifting (Asparouhov & Muthén, 2021). See S2.2 for details on the manual BCH method. As an index of effect sizes, the difference between the standardized scores of the distal outcomes (*z* scores) and were interpreted as small (.20), moderate (.50), and strong (.80) mean differences. Analytical codes for the analyses are available in S3 of the Supplemental Materials.

#### Variable-Oriented Approach: Relative Weight Analysis

To examine the unique contribution of each of the motivational variables on T1 and T2 exploration and identity commitment, we conducted relative weight analyses (RWA). RWA tests the relative importance of each of the predictor variables and ranks them based on their contribution to the variance explained in the outcome variable (i.e., *R*^2^), while accounting for the correlated nature of predictors to circumvent issues of multicollinearity (Tonidandel & LeBreton, 2011). This analysis allows us to identify the “key motivational drivers” in youth’s career development processes.

## Results

Measurement models conducted as part of preliminary analysis (for all study variables, including longitudinal invariance testing) showed an acceptable to excellent fit to the data. Factor scores were obtained from these models to be used in the main analysis. Table 1 presents descriptive statistics and correlation matrix of the factor scores (see Section S1 of the Online Supplements for detailed results).

**Table 1.** Correlations, Means, and Standard Deviations for Estimated Latent Variables.

	SE	IM	ID	IJ	EX	Explo1	Explo2	Ident1	Ident2	Girls^a^	Age^a^	Grades^a^	SFAI^a^
IM	**0.45**												
ID	**0.44**	**0.38**											
IJ	−0.08	−0.04	−0.01										
EX	**−0.17**	**−0.12**	**−0.25**	**0.48**									
Explo1	**0.52**	**0.39**	**0.18**	−0.01	−0.06								
Explo2	**0.41**	**0.34**	**0.20**	0.01	−0.15	**0.49**							
Commit1	**0.42**	**0.15**	**0.14**	**−0.14**	**−0.13**	**0.38**	0.19						
Commit2	**0.35**	**0.16**	**0.18**	−0.13	**−0.26**	**0.26**	**0.34**	**0.66**					
Girls^a^	0.09	**0.16**	0.06	0.06	**−0.14**	**0.12**	**0.21**	−0.07	−0.09				
Age^a^	0.10	0.07	0.09	−0.03	−0.06	0.09	0.09	0.03	−0.03	**−0.09**			
Grades^a^	0.08	−0.02	0.12	0.02	−0.11	−0.03	−0.03	0.06	0.05	**0.15**	**−0.30**		
SFAI^a^	0.09	0.02	0.02	−0.07	−0.09	−0.09	−0.09	−0.09	0.02	0.01	**0.14**	**−0.11**	
*M*	3.74	4.82	5.97	2.89	2.35	3.26	3.28	3.47	3.47	0.54	14.24	75.96	0.09
*SD*	0.63	1.60	1.03	1.68	1.50	0.84	0.93	1.00	1.08	0.50	0.51	9.83	0.19

*Note.* SE = self-efficacy; IM = intrinsic motivations; ID = identified regulation; IJ = introjected regulation; EX = external regulation; Explo = in-depth exploration; Commit = vocational identity development; Grades = school grades; SFAI = socio-familial adversity index. Bolded estimates are statistically significant (*p* < .05). These estimates for latent variables were obtained using the effects-coding method that preserves the original scales (Little et al., 2006).

^a^Variables with an asterisk (*) are observed (not latent) variables.

### Person-Oriented Approach: Profiles of Career Decision-Making Self-Efficacy and Autonomy

**Figure 1.**
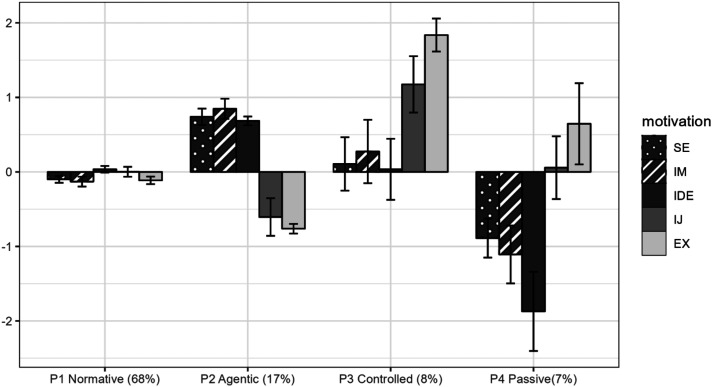
Final 4-Profile Solution of Motivations in Career Decision-Making (*N* = 637). *Note*. P1–P4 = Profile 1–Profile 4; SE = self-efficacy; IM = intrinsic motivation; IDE = identified regulation, IJ = introjected regulation; EX = external regulation. The error bars denote the 95% confidence intervals. The indicators are factor scores saved from measurement models where the latent mean is set to 0 and latent variance, approximating 1.

Although the aLMR and BLRT pointed to a 3-profile solution, all the other indices kept decreasing with the addition of a profile (see Table S5 for the fit indices of the 1- to 8-profile solutions). An elbow plot (in Figure S1) showed the presence of a plateau around the 4-profile solution. Upon inspecting the profiles’ interpretability and meaning, the 4-profile solution was retained. This solution showed a good level of classification accuracy (entropy = .92). Figure 1 shows the bar graph of the profile means (see Figure S2 for a line graph and Table S6 for profile means).

As shown in Figure 1, Profile 1 (68% of the sample) included youth reporting average levels of self-efficacy and all four types of motivations along the autonomy continuum. This profile was labeled “Normative” because it groups participants whose self-efficacy and autonomy scores were close to 0, which represents scores approximating the sample’s average (due to the use of factor scores). Profile 2 (17%) included youth reporting high levels of self-efficacy, intrinsic motivation, and identified regulation, and low levels of introjected and external regulation. This profile was labeled “Agentic” because these youth were not only self-efficacious in their career decision-making but also were driven by interests and personal values, and not by internal or external pressures. Profile 3 (8%) included youth reporting very high levels of introjected and external regulations, and average levels of self-efficacy, intrinsic motivation, and identified regulation. This profile was labeled “Controlled” because, despite their average scores on autonomous motivations and self-efficacy, they reported a high level of controlled motivations. Profile 4 (7%) included youth reporting low levels of self-efficacy, intrinsic motivations, and identified regulation, and above-average levels of external regulations. This profile was labeled “Passive” because these youth showed substantially low levels of autonomous motivations and self-efficacy, neither were they motivated by controlled reasons.

#### Mean-Level Differences in Exploration and Commitment across Profiles

Table 2 presents profile-specific means of career exploration and commitment. Pairwise comparisons showed that youth belonging to the Agentic profile reported the highest levels of exploration and commitment both at T1 and T2. The differences were large for comparisons with Normative and Passive profiles, and moderate for comparisons with the Controlled profile. As expected, those in the Passive profile reported the lowest levels of exploration and commitment at both timepoints. The effect size was moderate to large for all pairwise comparisons, except the comparison with the Controlled profile on T2 commitment, which yielded a small difference. Compared to the Normative youth, those in the Controlled profile explored more at T1 (a small difference) but they showed weaker identity commitment at T2 (a small to moderate difference).

**Table 2.** Profile-specific Means and Pairwise Comparisons of Exploration and Identity (*N* = 637).

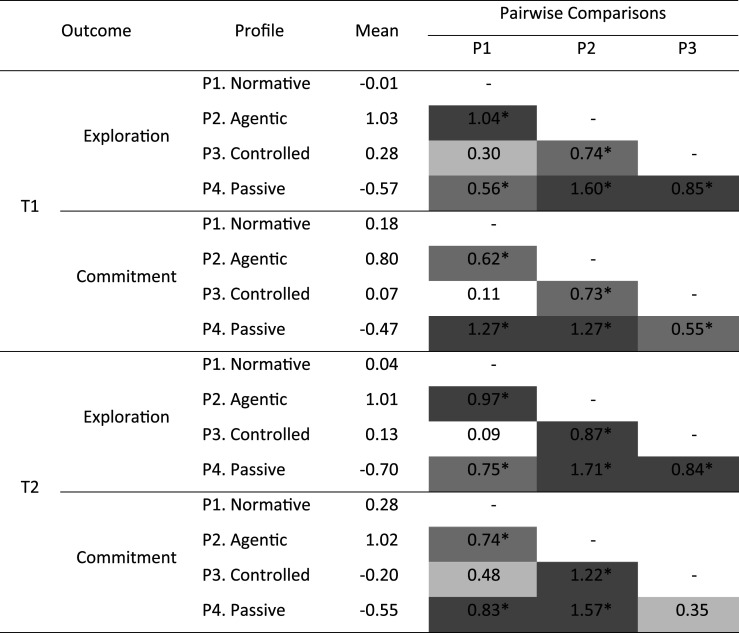

*Note.* P1–P4 = Profiles 1–4. T1 or Time 1 corresponds to the last year of high school; T2 or Time 2 corresponds to the year following the postsecondary transition. The mean estimates represent z scores (*M =* 0, *SD* = 1), the difference of which can be interpreted as .20 (small), .50 (moderate), and .80 (large). The shades indicate the effect sizes, with darker shades indicating a strong effect. The mean estimates were obtained, while controlling for socio-familial adversity, school grades, and gender. Differences between profiles are indicated by Cohen’s *d*, which can *p < .05, *p* < .05.

### Variable-Oriented Approach: Relative Importance of Motivational Variables

Table 3 reports the results of the RWA conducted on career development, while controlling for potential confounders, including gender, age, school grades, and SFAI. Beginning with T1 (pre-transition) outcomes, self-efficacy was the strongest predictor of both exploration and commitment, accounting for staggering 65% and 78% of the explained variance. Intrinsic motivation also made unique, substantial contribution particularly to exploration (26%), but much weakly to commitment (6%). Identified regulation explained roughly 5% of the variance in exploration and commitment, playing a less central role. Controlled motivations (introjected and external) contributed very little unique information to exploration (less than 1%), while bearing detrimental implications for commitment (−4% and −3%, respectively) as indicated by their negative signs. As for T2 (post-transition) outcomes, similar to the T1 results, self-efficacy remained the strongest predictor (55% and 58%), followed by intrinsic motivation, contributing 30% of the explained variance in exploration, albeit much smaller variance explained (6%) in commitment, with identified regulation playing a less central role (5% and 9%). One key difference that emerged at T2 is that external regulation explained much larger variance in exploration and particularly so in commitment (−5% and −20%), highlighting the potential long-term implication of external regulation on youth vocational identity development.

**Table 3.** Relative Weights Analysis of Motivation Predicting Exploration and Commitment.

Predictors	Before the Transition				After the Transition			
	Exploration		Commitment		Exploration		Commitment	
	RW	%	RW	%	RW	%	RW	%
Motivational variables								
Self-efficacy	.28	65.73	.21	77.55	.22	54.67	.17	57.52
Intrinsic	.11	25.83	.02	6.15	.12	30.33	.02	6.12
Identified	.02	4.99	.02	5.51	.02	5.39	.03	9.19
Introjected	.00	0.13	.01	−4.29	.01	1.62	.01	−4.31
External	.00	−0.55	.01	−3.33	.02	−5.27	.06	−20.24
Statistical controls								
Gender^a^	.01	1.29	.00	−1.60	.01	1.90	.01	−2.01
Age	.00	0.76	.00	0.08	.00	0.41	.00	−0.08
School grades	.00	−0.13	.00	0.40	.00	−0.13	.00	0.20
SFAI	.00	−0.58	.00	−1.09	.00	−0.29	.00	−0.33
*R* ^*2*^	.43		.27		.40		.29	

*Note.* RW is an estimated *R*^*2*^ (explained variance) associated with each predictor. % is the same relative weight converted to a percentage of total *R*^*2*^ such that they sum up to 100. SFAI = socio-familial adversity index score (calculated using the measures of family type, household income, and parental educational level).

^a^boys = 0; girls = 1.

## Discussion

The purpose of this study was to contribute to a more holistic understanding of youth agency in career development by examining career decision-making *self-efficacy* grounded in the social cognitive tradition (Bandura, 2005; Lent & Fouad, 2010) and career decision-making *autonomy* grounded in the self-determination theory (Ryan & Deci, 2017; Soenens & Vansteenkiste, 2011) as motivational precursors to in-depth career exploration and identity commitment. Using person- and variable-oriented analytical strategies and involving a stratified sample French-Canadian youth, we examined (1) heterogeneity in the pattern by which self-efficacy and autonomy components combine within individuals, (2) differences between these subgroups on exploration and commitment, both before and after the postsecondary transition, and (3) the relative importance of each of the motivational variables in predicting career development.

As expected, we identified four subgroups of youth based on the combinations of self-efficacy, intrinsic and identified regulations (autonomous motivations), and introjected and external regulations (controlled motivations). The largest group is the normative group of youth (68% of the sample), who scored average in all indicators, thereby serving as a reference profile for comparisons. The three other groups include the agentic youth (17%; high self-efficacy and autonomous motivations), controlled youth (8%; average levels of self-efficacy and autonomous motivations, but extremely high controlled motivations), and passive youth (7%; low self-efficacy and autonomous motivations, high external regulation). Unsurprisingly, agentic youth reported the highest level of exploration and identity, compared to their peers both before and after the transition, extending previous research demonstrating unique contributions of self-efficacy and autonomy in predicting career indecision among college students (Guay et al., 2003). Controlled youth, however, despite having engaged in greater exploration before the transition, saw a drop in identity after they had undergone the transition, a finding concurring with and extending previous cross-sectional studies examining the import of controlled and autonomous motivations on career development among high school students (Paixão & Gamboa, 2017, 2021). The most worrisome, passive youth explored the least and reported fuzzier identity across the transition. This profile of youth may be comparable to the 10% of adolescents in Christiaens et al. (2021)’s study, who did not seem to commit to any identity and remained unengaged in identity work across all 8 years spanning the secondary and postsecondary education. These youth characterized by low motivation and low engagement in identity processes may be at a higher risk for psychosocial problems (Branje et al., 2021) or for ending up in an unsatisfying career; hence, special attention needs to be geared toward identifying and intervening with these youth.

Complementing these findings, relative weight analyses showed that self-efficacy was the strongest predictor of exploration and identity, explaining more than half of the variances in career exploration and identity both before and after the transition. These results lend support to the social cognitive career theory (Lent & Fouad, 2010), suggesting self-efficacy as a key and immediate precursor of adolescent career development. Nevertheless, it is important to highlight that intrinsic motivation added substantial, albeit weaker, contribution, explaining over a quarter of the variance in exploration at both timepoints, above and beyond what is explained by self-efficacy. Of note, external regulation (being motivated by rewards and punishment) had an undermining implication for identity, a finding that became evident only after the postsecondary transition. One interpretation is that some youth may engage in exploration because of internal and external pressures, especially when the developmental deadline of the postsecondary transition is imminent. These pressured youth may engage in suboptimal forms of career exploration and commit to a career identity that does not align with one’s authentic self (Soenens & Vansteenkiste, 2011; Waterman, 2015).

### Theoretical and Practical Implications

Our findings have implications for motivational theories. Despite self-efficacy and autonomous motivations being distinct constructs, our findings highlight that these two motivational phenomena often occur simultaneously, and this motivational configuration provides a basis for youth’s optimal functioning in career development. That is, when youth feel confident and volitional, guided by their personal interests and values and unhindered by internal or external pressures, they take agency and ownership in their career development—to explore the world of work and self-reflect to eventually commit to a career identity that aligns with the self (e.g., values, interests, goals, and needs; Soenens & Vansteenkiste, 2011; Waterman, 2015).

At the same time, our findings provide a nuanced understanding of what it means to be “agentic,” by showing instances in which feeling confident may be accompanied by different levels of controlled motivations. The case of controlled youth in our study—who showed greater motivation in quantity but of suboptimal quality—illustrates the importance of examining quality—not just the quantity—of motivations, in line with SDT’s perspective (Ryan & Deci, 2017; Soenens & Vansteenkiste, 2011). Pressure, imposed by themselves, others (e.g., parents and others), or the developmental deadlines, has the potential to motivate youth toward career development activities. However, once the urgency has been lifted, negative consequences associated with controlled motivation are likely to surface, interfering with identity consolidation and integration.

These findings have practical implications. School counselors, parents, and teachers need to pay special attention to how they are motivating youth to navigate their career path—by supporting youth’s agency through autonomy-supportive and structuring practices, while minimizing controlling strategies. Practices promoting career decision-making self-efficacy well documented in meta-analyses include using workbooks and written exercises, gathering information about the world of work, and providing individualized feedback about tests results, career goals and plans (Brown, 2017; Whiston et al., 2017). To support youth’s autonomous functioning, socializing agents need to prioritize youth’s perspective and feelings, accept them as they are without trying to change them, and encourage self-expressions, while avoiding controlling practices like using rewards and punishment, imposing performance pressures, and inducing guilt or shame (Ahn et al., 2022).

In light of results from the latent profile analyses that self-efficacy and autonomous motivations go hand in hand, it is possible that there is a sequential process in the development of agency. As suggested by previous research (Cordeiro et al., 2015; Guay et al., 2006; Zhang et al., 2019), self-efficacy may be a more immediate precursor of exploration and commitment, whereas autonomy plays a more distal role in initiating and directing exploratory behaviors, which in turn helps develop career decision-making self-efficacy. Future studies are needed to examine the time-sensitive and unique role of self-efficacy and autonomy in adolescents’ career development. Another interesting avenue of research for motivational literature is to understand the role of autonomy in the development of self-efficacy.

Lastly, our findings highlight heterogeneity observed in the transitional periods, which can be a period of stress and challenge for some, as well as a period of identity development and psychological growth for others (Branje, 2022; Christiaens et al., 2021; Marttinen et al., 2018). In particular, the small yet non-negligible proportion of youth in controlled and passive profiles may be of concern to parents and educators. At the same time, the salient role played by external regulation in the post-transition identity development reiterates the need for more longitudinal studies focusing on transitional periods in the study of career development (Branje, 2022; Marttinen et al., 2018). Because the period under study is one of the two major postsecondary transitions in Quebec’s context, with the second one entailing moving from college to university or job market, how youth navigate this first transition may hold the key to how they prepare for the second transition (toward higher education or workforce).

### Strengths and Limitations

The present study examined identity processes before and after the transition out of high school and illustrated the long-term implication of considering career decision-making autonomy, a finding that may otherwise be masked in cross-sectional studies. Also, by casting career development through an integrated motivational lens and conceptualizing agency as arising from as perceived competence and autonomy, the present study aimed to expand the understanding of how to foster the sense of agency among youth. Lastly, the use of the person- and variable-oriented data analyses allows us to examine the unique and joint contributions of these motivational resources to career development.

Despite these strengths, some methodological aspects of the study limit the findings’ generalizability. The first pertains to the representativeness of the sample. Most of the participants were college-attending and university-bound; and they tended to perform better at school and come from higher SES than those lost to attrition. Our findings, therefore, may not be generalizable to non-college-bound youth who join the job market after high school. Further research is needed to examine the career developmental processes of youths in different walks of life (e.g., non-college-bound youth and those from disadvantaged backgrounds). Second, this study relied on youth self-reports alone, which may be subject to single-informant bias. Future studies can include more objective evaluations of youth career development, such as by including multi-informants’ reports about youth functioning.

## Conclusions

Our findings suggest that feelings of both efficacy and autonomy are two key pillars of agency. When adolescents feel both autonomous and competent, perceiving little to no pressure, they approach their career development with a sense of agency and ownership, exploring their world of work and themselves then commit to a career identity that best reflects their self. However, being motivated by rewards and punishment in career development can be detrimental in the long term, particularly after the postsecondary transition once the situational and developmental pressure (of choosing a career path) has been lifted. Rather than framing transition points as a deadline by which all adolescents must complete their identity work, identity agents like parents and teachers would need to take the motivational perspective to support youth’s autonomy and self-efficacy toward a lifelong process of developing the self.

## Supplemental Material

Supplemental Material - Motivational Resources of Agency in Adolescents’ Career Development in Postsecondary Transition: More than Being Self-Efficacious Supplemental Material for Motivational Resources of Agency in Adolescents’ Career Development in Postsecondary Transition: More than Being Self-Efficacious by Jiseul Sophia Ahn, Catherine F. Ratelle, Stéphane Duchesne, and André Plamondon in Journal of Career Development.

## Note

1. Despite some theoretical divergence in social cognitive theory’s self-efficacy (i.e., cognitive judgment of one’s capacity) and SDT’s competence (i.e., a basic need to be satisfied), both pertain to perceptions of one’s mastery of the environment and therefore the phenomenological experiences are quite blurred and if not indistinguishable, especially in self-reports (e.g., Sweet et al., 2012).
