# Supplementary material for: Motivational Resources of Agency in Adolescents’ Career Development in Postsecondary Transition: More than Being Self-Efficacious
Source: J Career Dev. 2025 Jun 14;52(4):449–68. doi: 10.1177/08948453251351576 (PMC12187008; doi:10.1177/08948453251351576)
Supplement: Supplemental Material - Motivational Resources of Agency in Adolescents’ Career Development in Postsecondary Transition: More than Being Self-Efficacious [file sj-pdf-1-jcd-10.1177_08948453251351576.pdf]

Online Supplemental Materials for

Motivational Resources of Agency in Adolescents' Career Development during Postsecondary Transition: More Than Being Self-Efficacious

Table of Contents

Section S1. Preliminary Measurement Models 2

1.1 Autonomy in Career Decision-Making Activities 2

1.2 Self-efficacy in Career Decision-Making Activities 3

Table S1. *Alternative Measurement Models of Self-efficacy* 3

Table S2. *Factor loadings on the General Factor in the Bifactor Model (M3 in Table S1)* 4

1.3 Measurement invariance 5

1.4 In-Depth Career Exploration 6

Table S3. *Testing Measurement Invariance of In-Depth Career Exploration* 6

1.5 Vocational Identity Commitment 7

Table S4. *Testing Measurement Invariance of Vocational Identity Commitment* 7

1.6 Socio-familial adversity index 8

Section 2. Main Analyses 9

2.1 Missing Data Estimation 9

2.2 Manual BCH method 9

Table S5. *Comparing Unconditional Latent Profile Models (1- to 8-Profile Solutions)* 10

Table S6. *Unstandardized Within-Profile Means of Motivations in Career Decision-Making* 11

Figure S1. *Elbow Plot the Information Criteria for the Latent Profile Analyses* 12

Figure S2. *Line Graph of the 4-Profile Solution of Motivations (N = 637)* 13

Figure S3. *Line Graph of the 4-Profile Solution of Motivations Using Effects-Coding (N = 637)* 14

Section 3. Mplus Syntax 15

Syntax 1. *Latent Profile Analysis (Unconditional Model)* 15

Syntax 2. Manual BCH method - Step 1 16

Syntax 2. Manual BCH method - Step 3 18

Syntax 4. *Mediation Model* 20

Section S1. Preliminary Measurement Models

1.1 Autonomy in Career Decision-Making Activities

To account for the method factors associated with each career decision-making activity, correlations among residual variances of items describing the same activity were allowed. Items assessing autonomy in two career decision-making activities (seeking career information on careers and on study programs) showed very high correlations (*r*s > .73). So, four items representing 'seeking information on study programs' were excluded in the measurement model. Each type of motivation (e.g., intrinsic motivation) was modeled as a latent variable indexed by seven items (one item from each of the seven career decision-making activities). The measurement model yielded an excellent fit to the data, χ^2 (302) = 520.186, $p < .001$, CFI = .962, RMSEA = .034, SRMR = .048, and excellent internal consistency for all four motivation types (ωs = .91–.95).

1.2 Self-efficacy in Career Decision-Making Activities

Table S1 shows the model fits of the hypothesized and alternative measurement models we tested in this study. Neither a 1- nor a 5-factor model, however, yielded a satisfying fit, a finding previously reported (Betz et al., 1996; Creed et al., 2002; Hampton, 2005). Therefore, we selected 8 items with a factor loading of .60 or above on a general factor in a bifactor modeling (Török et al., 2016) and then used them to model self-efficacy as a latent variable. Table S2 shows the factor loadings of the retained items. The measurement model with retained items yielded an excellent fit to the data, $\chi^2(20) = 56.116$, $p < .001$, CFI = .949, RMSEA = .053, SRMR = .043, and satisfactory internal consistency (ω = .85). For model parsimony, items were aggregated to form three parcels (of 3, 2, and 2 items respectively), which were used as indicators in the just-identified measurement model of self-efficacy.

Table S1. *Alternative Measurement Models of Self-efficacy*

Models	χ^2	df	CFI	RMSEA	SRMR
M1. 1-factor CFA with 25 items	963.927	275	.769	.063	.071
M2. 5-factor CFA with 25 items	795.605	265	.821	.070	.065
M3. Bifactor CFA with 25 items	669.705	250	.859	.061	.051
M4. 1-factor CFA with 8 items[a]	56.817	20	.951	.054	.043

Note.[a] The 8 items were chosen from M3 if the factor loadings on the general factor were greater than .60.

Table S2. *Factor loadings on the General Factor in the Bifactor Model (M3 in Table S1)*

Item	Label	λ_g
1	Finding information in the library	0.45
2	Select one college major from a list of potential majors you are considering	0.58
3	Make a plan of your goals for the next five years	0.64
**4**	**Determine the steps to take if you are having trouble with your chosen college major or job**	**0.67**
5	Accurately rate your abilities	0.58
6	Select on occupation from a list of potential occupations you are considering	0.55
**7**	**Determine the steps you need to take to be successful in your chosen major or occupation**	**0.71**
8	Continue to work at your major or career goal even when you get frustrated	0.57
9	Determine what your ideal job would be	0.53
10	Find out the employment trends for an occupation over the next ten years	0.56
11	Choose a career that will fit with the lifestyle you want to have	0.51
12	Prepare a good resume	0.55
13	Change college majors if you do not like your first choice	0.50
14	Decide what is most important to you about an occupation	0.63
15	Find out about the average yearly earnings of people in an occupation	0.39
16	Make a career decision and then not worry about whether it was right or wrong	0.47
17	Change occupations if you are not satisfied with the one you enter	0.47
**18**	**Figure out what you are and are not ready to sacrifice to achieve your career goals**	**0.63**
19	Talk with a person already employed in the field you are interested in	0.56
20	Choose a college major or a job that will fit your interests	0.56
**21**	**Identify employers, firms, and institutions relevant to your career possibilities**	**0.60**
22	Define the type of lifestyle you would like to live	0.51
23	Find information about getting further education/training in a occupation of interest to you	0.58
**24**	**Successfully manage the job interview process**	**0.61**
**25**	**Identify some reasonable major/career alternatives if you are unable to get your first choice**	**0.64**

Note. The bolded items have factor loadings on the general self-efficacy factor (λ_g) greater than .60. They are items selected for M4 in Table S1.

1.3 Measurement invariance

Testing the measurement invariance in longitudinal studies is a recommended practice because it ensures that the meaning of the constructs does not change over time and are comparable across waves (Meredith & Horn, 2001). Invariance testing proceeded in four steps.

1. *Configural invariance* tests if the model has the same factor structure across time. It involves estimating the same baseline model across waves. If the overall fit of the model was good, the configural invariance was accepted, then we proceeded to testing metric invariance.
2. *Metric invariance* tests if the factor loadings remain equivalent across time. It involves imposing equality constraints on the factor loadings of the same indicators across waves. When metric invariance was accepted—equality constraints did not bring a significant loss in model fits ($\Delta\chi_{SB}^2$, $p < .05$)—, we proceeded to testing scalar invariance.
3. *Scalar invariance* tests if any mean differences in the shared variance of the indicators are captured by the mean differences in the latent construct. It involves imposing equality constraints on the intercepts of the same indicators across waves. When the scalar invariance was accepted, we proceeded to testing strict invariance.
4. *Strict invariance* tests if the variance unexplained for by the latent factor (due to measurement errors and randomness) is equal across time. It involves imposing equality constraints on the residual variances of the indicators.

Each level of invariance testing was considered accepted if the loss in model fit is smaller than .010 for CFI and .015 for RMSEA (Chen, 2007). We tested the longitudinal invariance of measurement models of (1) in-depth exploration and (2) vocational identity. Table S3 presents the measurement invariance testing for all 4 models. All models reached the strict invariance.

1.4 In-Depth Career Exploration

Out of the 15 items in the original scale, 7 items were selected based on their conformity to the conceptual definition of in-depth career exploration. Opinions were solicited from two experts in career development, one of whom was external to this study's authorship. The selected items are as follows:

1. I obtained information on specific jobs or companies related to the field of work that I might choose.
2. I initiated conversations with knowledgeable individuals in my career area.
3. I obtained information on the labor market and general job opportunities in my career area.
4. I sought information on specific areas of career interests.
5. I reflected on how my past integrates with my future career.
6. I understood the relevance of my past behaviors for my future career.
7. I have been retrospective in thinking about my career.

The first four items (1-4) belong to the dimension of environment exploration, whereas the next three items (5-7) belong to the dimension of self-exploration. Given the multidimensional aspect of our scale, we created three domain-representative parcels whereby items from each dimension (self and environment exploration) are randomly assigned to a parcel in such a way that dimension-specific variance is equally distributed across parcels and that each parcels represents the whole "domain" of the multidimensional construct (Little et al., 2013). Table S4 compares the fit indices for each level of measurement invariance.

Table S3. *Testing Measurement Invariance of In-Depth Career Exploration*

Models	χ^2	*df*	*p*-value	CFI	RMSEA	SRMR	ΔCFI	ΔRMSEA	Decision
M1. Configural Invariance	73.558	51	0.021	0.987	0.026	0.043	-	-	-
M2. Metric Invariance	79.943	58	0.0297	0.987	0.024	0.048	0	-.002	Accept
M3. Scalar Invariance	91.104	65	0.018	0.984	0.025	0.05	-.003	+.001	Accept
M4. Strict Invariance	98.372	72	0.0212	0.984	0.024	0.057	0	-.001	Accept

Note. df = degrees of freedom; CFI = comparative fit index; RMSEA = root mean square error of approximation; SRMR = standardized root mean squared residual; Δ = change in fit.

1.5 Vocational Identity Commitment

Out of 18 items in the original scale, only 9 items were completed by participants across waves; hence, the measurement models were estimated with 9 items only. They are as follows:

1. If I had to make an occupational choice right now, I'm afraid I would make a bad choice.
2. Making up my mind about a career has been a long and difficult problem for me.
3. I am confused about the whole problem of deciding on a career.
4. I am not sure that my present occupational choice or job is right for me.
5. I don't know enough about what workers do in various occupations.
6. No single occupation appeals strongly to me.
7. I am uncertain about which occupation I would enjoy.
8. I am not sure of myself in many areas of life.
9. I can't understand how some people can be so set about what they want to do.

The items were aggregated to form three parcels for the measurement model. This model demonstrated longitudinal invariance and yielded an excellent fit to the data, $\chi2(14) = 18.77$, $p = .174$, CFI = .996, RMSEA = .023, SRMR = .079, with excellent internal consistency ($\omega = .93$). Table S4 compares the fit indices for each level of measurement invariance.

Table S4. *Testing Measurement Invariance of Vocational Identity Commitment*

Models	χ^2	*df*	*p*-value	CFI	RMSEA	SRMR	ΔCFI	ΔRMSEA	Decision
M1. Configural Invariance	8.334	5	0.1388	0.997	0.032	0.029	-	-	-
M2. Metric Invariance	12.227	8	0.1414	0.997	0.029	0.073	0	-.003	Accept
M3. Scalar Invariance	13.504	11	0.2617	0.998	0.019	0.076	+.001	-.010	Accept
M4. Strict Invariance	8.334	5	0.1388	0.997	0.032	0.029	-.001	+.013	Accept

Note. df = degrees of freedom; CFI = comparative fit index; RMSEA = root mean square error of approximation; SRMR = standardized root mean squared residual; Δ = change in fit.

1.6 Socio-familial adversity index

To parsimoniously account for the socio-familial factor, we calculated a socio-familial adversity index (SFAI), using the measures of family type, household income, and parental educational level where each of the three indicators were conceptualized in terms of risk, with 0 indicating not at risk and 1 indicating at risk (Ratelle et al., 2020). Family type was scored as 0 for families with two-biological parents (79% of the sample); as 1 for other family types (21%). Household income was scored as 1 for families with an annual income lower than $29,999 CAD (7%); as 0 if income is higher than $29,999 CAD (93%). Lastly, parental educational level was scored as 1 if parents did not obtain a high school diploma (5%); as 0 if parents had a high school diploma at the minimum (95%). These binary variables were then averaged to create an SFAI, ranging from 0 to 1.

Section 2. Main Analyses

2.1 Missing Data Estimation

To deal with missing data, we used the full information maximum likelihood (FIML) estimation with robust standard errors (the MLR option in Mplus 8.1), under the missing at random assumption. FIML is superior to the listwise deletion method because it uses all available data to calculate a likelihood function for each case, thus improving statistical power, while correcting for nonnormality. Furthermore, to improve the missing data estimation, we used the principal component approach (Howard et al., 2015) to extract seven principal components explaining 40% of the variance in 70+ covariates (e.g., sociodemographic and adjustment variables assessed at the onset). These components were then integrated as auxiliary variables in all model estimations to make the missing at random assumption more plausible and to improve statistical power (Graham, 2003).

2.2 Manual BCH method

The BCH method is recommended when estimating profile-specific means for continuous distal outcomes, because (1) this method uses “BCH weights” to account for probabilities of profile classification and; (2) it prevents profile shifting, a phenomenon occasionally observed when distal outcomes are integrated into the unconditional model (Asparouhov & Muthén, 2021). The manual 3-step BCH procedure involved, first, saving BCH weights from the best-fitting unconditional model and then specifying the auxiliary model where the mean levels of distal outcomes are estimated, while controlling for other covariates (Nylund-Gibson et al., 2019). The factor scores of the T1 and T2 scores of exploration and commitment were obtained from the most invariant measurement model, standardized into z scores (for interpretability), and then integrated into the final solution. The model estimates from the unconditional solution were fixed to their starting values to prevent profile shifting.

Table S5. *Comparing Unconditional Latent Profile Models (1- to 8-Profile Solutions)*

Profile	AIC	BIC	aBIC	Entropy	VLMR	aLMRT	BLRT
1	7885.427	7929.994	7898.245				
2	7507.725	7579.033	7528.234	0.962	0.000	0.000	0.000
3	7250.959	7349.008	7279.160	0.952	0.266	0.272	0.000
4	6956.339	7081.129	6992.231	0.909	0.016	0.018	0.000
5	6796.185	6947.715	6839.768	0.926	0.183	0.188	0.000
6	6679.482	6857.753	6730.756	0.923	0.459	0.467	0.000
7	6572.073	6777.085	6631.038	0.963	0.212	0.215	0.000
8	6459.808	6691.560	6526.464	0.953	0.632	0.640	0.000

NOTE. AIC = Akaike information criteria; BIC= Bayesian information criteria; aBIC = sample-size adjusted BIC; VLMR = Vuong-Lo_Mendell-Rubin LRT p-value; aLMR = Lo-Mendell-Rubin adjusted LRT p-value; BLRT = the Bootstrapped Likelihood Ratio Test p-value.

Table S6. *Unstandardized Within-Profile Means of Motivations in Career Decision-Making*

Profile	Means (Standard Errors)				
	SE	IM	IDE	IJ	EX
P1. Normative	-0.08 (0.02)	-0.18 (0.05)	0.04 (0.02)	0.00 (0.04)	-0.13 (0.03)
P2. Agentic	0.59 (0.04)	1.23 (0.10)	0.69 (0.03)	-0.73 (0.16)	-0.89 (0.04)
P3. Controlled	0.08 (0.14)	0.38 (0.30)	0.03 (0.19)	1.38 (0.22)	2.09 (0.13)
P4. Passive	-0.74 (0.10)	-1.6 (0.27)	-1.89 (0.25)	0.08 (0.24)	0.75 (0.29)

Note. SE = self-efficacy; IM= intrinsic motivation; IDE = identified regulation, IJ = introjected regulation; EX = external regulation. The error bars denote the 95% confidence intervals.

Figure S1. *Elbow Plot the Information Criteria for the Latent Profile Analyses*

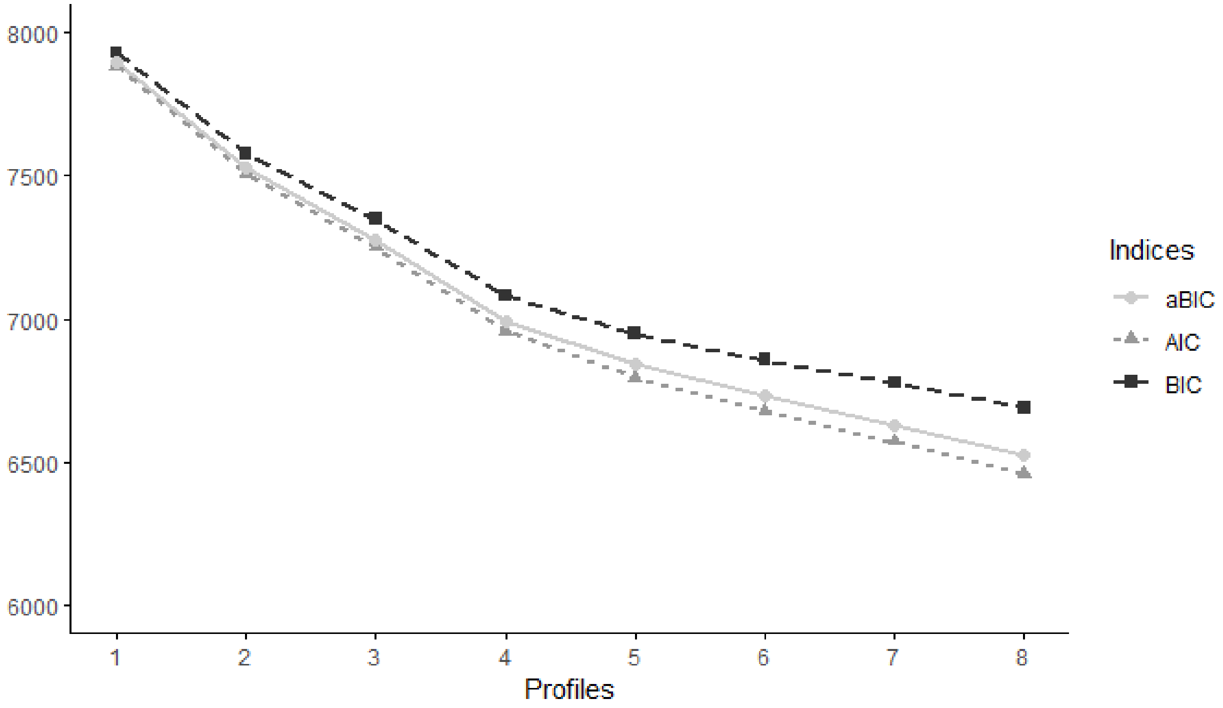

Note. AIC = Akaike information criteria; BIC= Bayesian information criteria; aBIC = sample-size adjusted BIC.

Figure S2. *Line Graph of the 4-Profile Solution of Motivations (N = 637)*

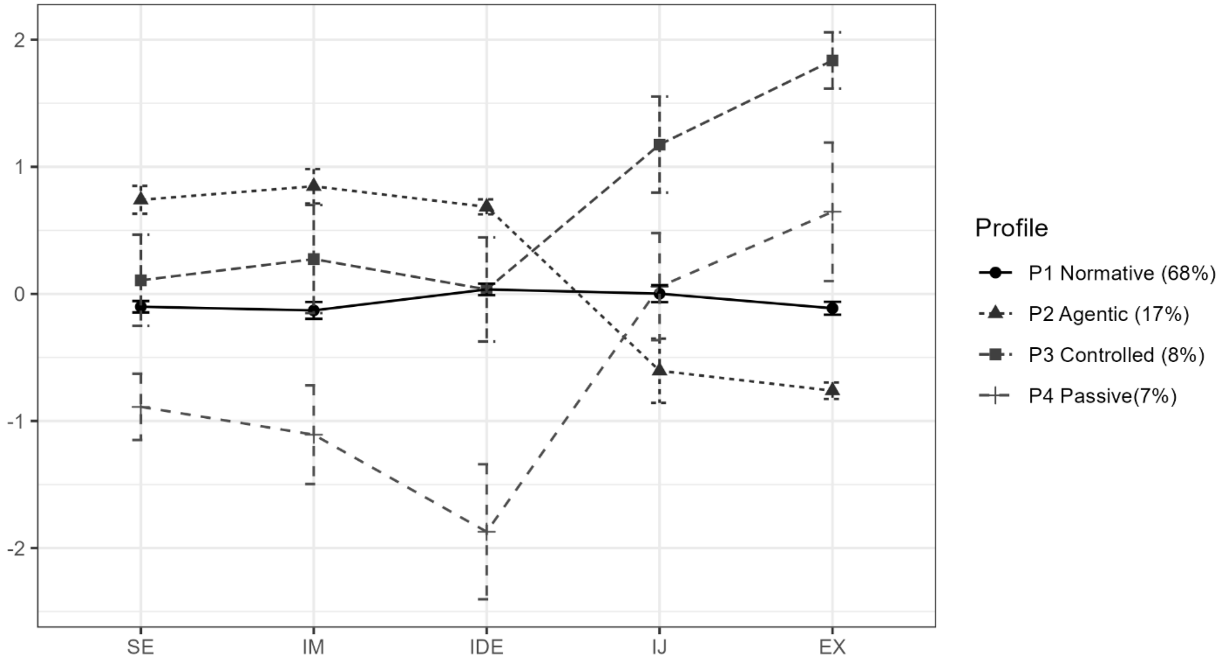

Note. SE = self-efficacy; IM= intrinsic motivation; IDE = identified regulation, IJ = introjected regulation; EX = external regulation. The error bars denote the 95% confidence intervals.

Figure S3. *Line Graph of the 4-Profile Solution of Motivations Using Effects-Coding (N = 637)*

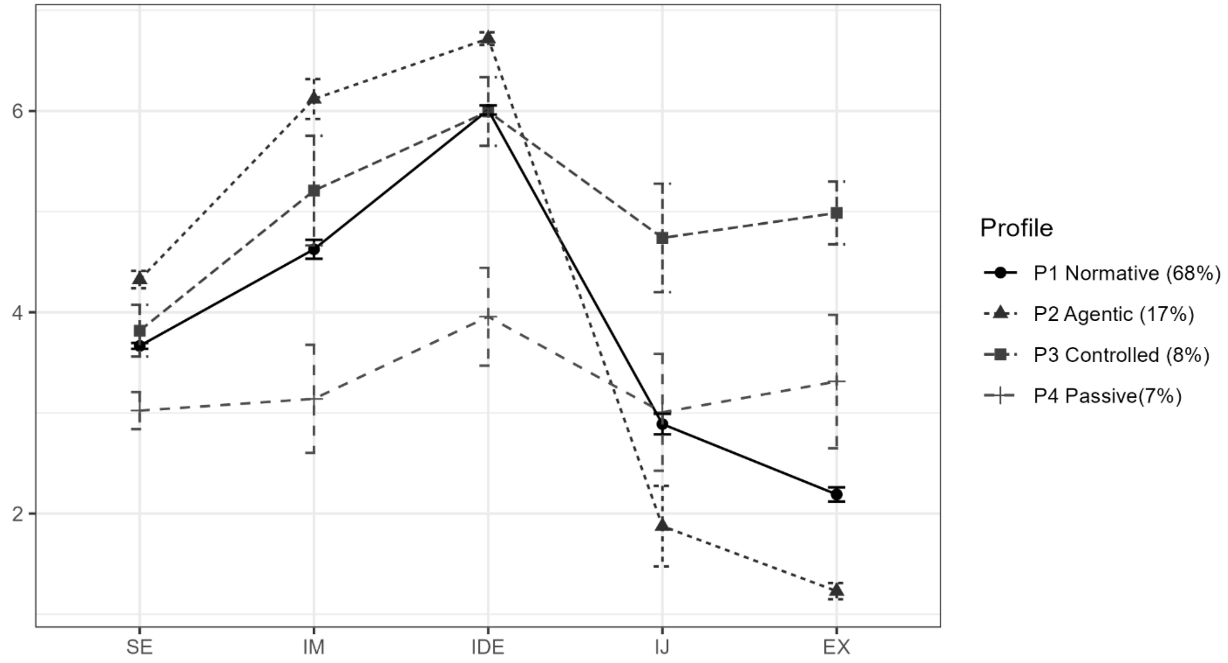

Note. SE = self-efficacy; IM= intrinsic motivation; IDE = identified regulation, IJ = introjected regulation; EX = external regulation. The error bars denote the 95% confidence intervals. Self-efficacy was assessed using a 5-point rating scale (1 to 5), while the four types of motivations along the autonomy continuum were assessed using a 7-point rating scale (1 to 7).

Section 3. Mplus Syntax

Syntax 1. *Latent Profile Analysis (Unconditional Model)*

```
VARIABLE:
NAMES = id se im ide ij ex explo1 explo2 idtt1 idtt2;
MISSING=.;
USEVARIABLES= se im ide ij ex;
CLASSES = c(4);

ANALYSIS:
TYPE = mixture;
ESTIMATOR = mlr;
PROCESS = 4;
STARTS=5000 300;
! requests 5000 sets of random svalues,
! with the best 300 of these starts retained for final stage
STITERATIONS = 200;
! requests that all random starts be allowed 200 iterations
LRTSTARTS = 0 0 500 200;
! STARTS options for tech14
! the first two digits for k-1 class model
! the last two for k class model

OUTPUT:
sampstat stdyx svalues tech4 tech7 tech11 tech14;
```

Syntax 2. Manual BCH method - Step 1

```
VARIABLE:
NAMES = id esexe sfai grade se im ide ij ex explo1 explo2 idtt1 idtt2;
MISSING=.;
AUXILIARY = explo1 explo2 idtt1 idtt2 sfai grade esexe;
USEVARIABLES= se im ide ij ex;
CLASSES = c(4)

ANALYSIS:
TYPE = mixture;
ESTIMATOR = mlr;
PROCESS = 4;
STARTS= 0;

MODEL:
     %OVERALL%

     [ c#1*-0.87809 ];
     [ c#2*1.35796 ];
     [ c#3*-0.73611 ];

     %C#1%

     [ se*-0.88872 ];
     [ im*-1.10751 ];
     [ ide*-1.87184 ];
     [ ij*0.05677 ];
     [ ex*0.64571 ];

     se*0.21825 (6);
     im*0.48080 (7);
     ide*0.21924 (8);
     ij*0.50082 (9);
     ex*0.24704 (10);

     %C#2%

     [ se*-0.10085 ];
     [ im*-0.13047 ];
     [ ide*0.03514 ];
     [ ij*0.00228 ];
     [ ex*-0.11283 ];

     se*0.21825 (6);
     im*0.48080 (7);
     ide*0.21924 (8);
     ij*0.50082 (9);
     ex*0.24704 (10);
```

```
    %C#3%

    [ se*0.10677 ];
    [ im*0.27370 ];
    [ ide*0.03473 ];
    [ ij*1.17500 ];
    [ ex*1.83710 ];

    se*0.21825 (6);
    im*0.48080 (7);
    ide*0.21924 (8);
    ij*0.50082 (9);
    ex*0.24704 (10);

    %C#4%

    [ se*0.73950 ];
    [ im*0.84725 ];
    [ ide*0.68500 ];
    [ ij*-0.60494 ];
    [ ex*-0.76240 ];

    se*0.21825 (6);
    im*0.48080 (7);
    ide*0.21924 (8);
    ij*0.50082 (9);
    ex*0.24704 (10);

OUTPUT:
sampstat stdyx svalues cinterval;

SAVEDATA:
    FILE IS bchweights.dat;
    SAVE=bchweights;
    MISSFLAG=-999;
```

Syntax 2. Manual BCH method - Step 3

```
VARIABLE:
NAMES =
     SE
     IM
     IDE
     IJ
     EX
     EXPLO1
     EXPLO2
     IDTT1
     IDTT2
     SFAI
     GRADE
     ESEXE
     BCHW1
     BCHW2
     BCHW3
     BCHW4   ;

MISSING=all(-999);
CLASSES = c(4);
TRAINING = bchw1-bchw4(bch);

DEFINE:
STANDARDIZE explo1 explo2 idtt1 idtt2;

ANALYSIS:

  TYPE = mixture;
  ESTIMATOR = mlr;

MODEL:

      %OVERALL%

     explo1 explo2 idtt1 idtt2 ON sfai grade esexe;
     [sfai grade esexe];

      %C#1%

     [explo1 explo2 idtt1 idtt2](d11 d21 d31 d41);
     explo1 explo2 idtt1 idtt2;

      %C#2%

      [explo1 explo2 idtt1 idtt2](d12 d22 d32 d42);
     explo1 explo2 idtt1 idtt2;
```

```
 %C#3%

 [explo1 explo2 idtt1 idtt2](d13 d23 d33 d43);
explo1 explo2 idtt1 idtt2;

 %C#4%

[explo1 explo2 idtt1 idtt2](d14 d24 d34 d44);
explo1 explo2 idtt1 idtt2;

MODEL CONSTRAINT:
    new(d11_d12 d11_d13 d11_d14
    d12_d13 d12_d14
    d13_d14
    d21_d22 d21_d23 d21_d24
    d22_d23 d22_d24
    d23_d24
    d31_d32 d31_d33 d31_d34
    d32_d33 d32_d34
    d33_d34
    d41_d42 d41_d43 d41_d44
    d42_d43 d42_d44
    d43_d44);

    d11_d12 = d11 - d12;
    d11_d13 = d11 - d13;
    d11_d14 = d11 - d14;
    d12_d13 = d12 - d13;
    d12_d14 = d12 - d14;
    d13_d14 = d13 - d14;

    d21_d22 = d21 - d22;
    d21_d23 = d21 - d23;
    d21_d24 = d21 - d24;
    d22_d23 = d22 - d23;
    d22_d24 = d22 - d24;
    d23_d24 = d23 - d24;

    d31_d32 = d31 - d32;
    d31_d33 = d31 - d33;
    d31_d34 = d31 - d34;
    d32_d33 = d32 - d33;
    d32_d34 = d32 - d34;
    d33_d34 = d33 - d34;

    d41_d42 = d41 - d42;
    d41_d43 = d41 - d43;
    d41_d44 = d41 - d44;
    d42_d43 = d42 - d43;
    d42_d44 = d42 - d44;
```

Syntax 4. *Mediation Model*

We only show syntaxes that differ from Syntax 3 in the MODEL section

```
! Measurement Model of SE
se BY se11-se13 ;

! Measurement Model of Autonomy
IM BY e3mvo4 e3mvo12 e3mvo16 e3mvo20 e3mvo24 e3mvo28 e3mvo32 (im1-im7);
ID BY e3mvo3 e3mvo11 e3mvo15 e3mvo19 e3mvo23 e3mvo27 e3mvo31 (id1-id7);
IJ BY e3mvo2 e3mvo10 e3mvo14 e3mvo18 e3mvo22 e3mvo26 e3mvo30 (ij1-ij7);
EX BY e3mvo1 e3mvo9 e3mvo13 e3mvo17 e3mvo21 e3mvo25 e3mvo29 (ex1-ex7);

! residual covariance between items belonging to the same decision-making task
e3mvo1-e3mvo4 WITH e3mvo1-e3mvo4;
e3mvo9-e3mvo12 WITH e3mvo9-e3mvo12;
e3mvo13-e3mvo16 WITH e3mvo13-e3mvo16;
e3mvo17-e3mvo20 WITH e3mvo17-e3mvo20;
e3mvo21-e3mvo24 WITH e3mvo21-e3mvo24;
e3mvo25-e3mvo28 WITH e3mvo25-e3mvo28;
e3mvo29-e3mvo32 WITH e3mvo29-e3mvo32;

! Predictors
se IM ID IJ EX explore1 identit1;
explore1 WITH identit1;

! Partial mediation model;

diff_explr ON se IM ID IJ EX explore1 identit1;
explore1 ON se IM ID IJ EX;
diff_iden ON se IM ID IJ EX identit1 explore1;
identit1 ON se IM ID IJ EX ;


MODEL INDIRECT:

diff_explr VIA explore1 se;
diff_explr VIA explore1 IM;
diff_explr VIA explore1 ID;
diff_explr VIA explore1 IJ;
diff_explr VIA explore1 EX;

diff_iden VIA identit1 se;
diff_iden VIA identit1 IM;
diff_iden VIA identit1 ID;
diff_iden VIA identit1 IJ;
diff_iden VIA identit1 EX;
```

References

Asparouhov, T., & Muthén, B. (2021). *Auxiliary Variables in Mixture Modeling: Using the Bch Method in Mplus to Estimate a Distal Outcome Model and an Arbitrary Secondary Model*. https://www.statmodel.com/examples/webnotes/webnote21.pdf

Betz, N. E., Klein, K. L., & Taylor, K. M. (1996). Evaluation of a Short Form of the Career Decision-Making Self-Efficacy Scale. *Journal of Career Assessment, 4*(1), 47-57. https://doi.org/10.1177/106907279600400103

Chen, F. F. (2007). Sensitivity of Goodness of Fit Indexes to Lack of Measurement Invariance. *Structural Equation Modeling: A Multidisciplinary Journal, 14*(3), 464-504. https://doi.org/10.1080/10705510701301834

Creed, P. A., Patton, W., & Watson, M. B. (2002). Cross-Cultural Equivalence of the Career Decision-Making Self-Efficacy Scale-Short Form: An Australian and South African Comparison. *Journal of Career Assessment, 10*(3), 327-342.

Graham, J. W. (2003). Adding Missing-Data-Relevant Variables to Fiml-Based Structural Equation Models. *Structural Equation Modeling: A Multidisciplinary Journal, 10*(1), 80-100. https://doi.org/10.1207/s15328007sem1001_4

Hampton, N. Z. (2005). Testing for the Structure of the Career Decision Self-Efficacy Scale-Short Form among Chinese College Students. *Journal of Career Assessment, 13*(1), 98-113. https://doi.org/10.1177/1069072704270298

Howard, W. J., Rhemtulla, M., & Little, T. D. (2015). Using Principal Components as Auxiliary Variables in Missing Data Estimation. *Multivariate Behavioral Research, 50*(3), 285-299. https://doi.org/10.1080/00273171.2014.999267

Meredith, W., & Horn, J. (2001). The Role of Factorial Invariance in Modeling Growth and Change. In L. M. Collins & A. G. Sayer (Eds.), *New Methods for the Analysis of Change.* (pp. 203-240). American Psychological Association. https://doi.org/10.1037/10409-007

Nylund-Gibson, K., Grimm, R. P., & Masyn, K. E. (2019). Prediction from Latent Classes: A Demonstration of Different Approaches to Include Distal Outcomes in Mixture Models. *Structural Equation Modeling: A Multidisciplinary Journal*, 1-19. https://doi.org/10.1080/10705511.2019.1590146

Török, R., Tóth-Király, I., Bőthe, B., & Orosz, G. (2016). Analyzing Models of Career Decision Self-Efficacy: First-Order, Hierarchical, and Bifactor Models of the Career Decision Self-Efficacy Scale. *Current Psychology, 36*(4), 764-773. https://doi.org/10.1007/s12144-016-9464-9
